# Veterans utilizing a federally qualified health center: a clinical snapshot

**DOI:** 10.1186/s40779-022-00379-y

**Published:** 2022-04-13

**Authors:** Thad E. Abrams, Bruce Alexander, Antonio Flores, M. Bryant Howren

**Affiliations:** 1 VHA Office of Rural Health, Veterans Rural Health Resource Center, Iowa City, IA 52246 USA; 2 Carver College of Medicine, The University of Iowa, Iowa City, IA 52242 USA; 3 Center for Access Delivery Research and Evaluation (CADRE), VA Iowa City Healthcare System, Iowa City, IA 52246 USA; 4 Community Health Centers of Southeastern Iowa, West Burlington, IA 52655 USA; 5 Department of Behavioral Sciences and Social Medicine, College of Medicine, Florida State University, Tallahassee, FL 32306 USA; 6 Florida Blue Center for Rural Health Research and Policy, College of Medicine, Florida State University, Tallahassee, FL 32306 USA

**Keywords:** Veterans, Federally qualified healthcare centers, Healthcare utilization, Dual use, Mental health

Dear Editor,

The Veterans Health Administration (VHA) provides healthcare for over 9 million enrolled veterans with approximately 2.7 million of those residing in rural areas [1]. The MISSION Act of 2018 emphasizes VHA collaboration with Federally Qualified Healthcare Centers (FQHC) to serve rural residing veterans and nearly all existing collaborations involve arrangement of payment for community-based care by VHA to FQHCs. Unfortunately, there is a paucity of descriptive clinical data on existing cross-system collaborations which may help characterize these veterans and aid understanding of conditions for which they may receive treatment across systems. Such data has implications for workforce training, development, and resource allocation [2]. The objective of this report is to describe different clinical profiles between two mutually exclusive samples: veterans engaged in FQHC only use, and VHA-enrolled veterans engaged in dual VHA and FQHC use.

The VHA Office of Rural Health supported a partnership between a Midwest VHA medical center and rural-based FQHC distant from the VHA aiming to systematically identify veterans presenting for care in the FQHC, screen for mental health issues, and initiate care coordination between organizations [3]. Veterans (*n* = 782) presenting for care in the FQHC were systematically identified at intake; the sample was then divided according to VHA utilization: (1) FQHC only use (*n* = 433, 55.4%), and (2) VHA and FQHC dual use (*n* = 349, 44.6%). Limited releases of information enabled access to each system’s administrative databases to obtain demographic characteristics and clinical diagnoses accordingly. All data presented here reflect patients presenting for care between January 1, 2018 to April 1, 2020.

Demographic characteristics by group are shown in Additional file 1: Table S1. The FQHC only group was younger and more often female. Diagnoses by International Classification of Diseases, Tenth Revision (ICD-10) codes revealed conditions frequently encountered in the veteran population with those most common including hypertension, lipid disorders, musculoskeletal disorders, cardiovascular disorders, anxiety disorders, depressive disorders, and diabetes (Table 1). Notably, relative to FQHC only use, dual users had significantly higher frequencies of post-traumatic stress disorder (PTSD), substance use, and sleep disorders; obesity, infectious diseases, and tobacco use disorders were significantly higher for FQHC only use veterans.

**Table 1:** ICD-10 diagnoses according to group [*n*(%)]*

Diagnosis**	FQHC use only veterans (*n* = 433)	Dual use veterans (*n* = 349)	*P*-value
Anxiety disorders	52 (12.0)	39 (11.2)	0.71
Depressive disorders	61 (14.1)	62 (17.8)	0.16
Post-traumatic stress disorder	14 (3.2)	37 (10.6)	< 0.0001
Substance abuse disorders	21 (4.8)	29 (8.3)	0.049
Cardiovascular disorder	71 (16.4)	71 (20.3)	0.15
Diabetes	58 (13.4)	45 (12.9)	0.83
Gastrointestinal disorders	78 (18.0)	67 (19.2)	0.67
Genitourinary disorders	76 (17.6)	47 (13.5)	0.11
Hypertension	144 (33.3)	106 (30.4)	0.39
Infectious disease	100 (23.1)	26 (7.4)	< 0.0001
Lipid disorders	96 (22.2)	92 (26.4)	0.17
Musculoskeletal/Joint disorders	126 (29.1)	103 (29.5)	0.89
Obesity	109 (25.2)	31 (8.9)	< 0.0001
Respiratory disease	89 (20.6)	60 (17.2)	0.23
Skin disorders non-bacterial	60 (13.9)	45 (12.9)	0.69
Sleep disorders	30 (6.9)	49 (14.0)	0.001
Tobacco use disorder	97 (22.4)	41 (11.7)	0.0001

*Comparisons were conducted using chi-square test with significance set at *P* < 0.05. Diagnosis codes were identified using only FQHC data for FQHC use only and VHA data for dual use veterans

**Diagnoses were classified using a modified version of the clinical classification software (CCS)®. Diagnoses codes are not mutually exclusive and individuals may have > 1 diagnoses in each category

*FQHC* Federally Qualified Healthcare Centers, *ICD-10* International Classification of Diseases, Tenth Revision

This study presents basic descriptive and clinical diagnosis information for two groups of veterans seeking care at VHA and/or a FQHC, respectively. It is also notable that a considerable number of veterans residing in a rural southeast portion of a Midwest state utilizing FQHC services maintained a relationship with VHA care despite a nearly a 60-min travel time.

Table 1 details diagnoses of PTSD, sleep disorders, and substance use conditions which were higher in dual use veterans relative to their FQHC only peers. This suggests that veterans may prefer treatment for these conditions in VHA, perhaps due to considerable mental and behavioral health resources (including expansive telehealth options) and policy mandates regarding wait times in VHA. Veterans may also choose between VHA and non-VHA resources based on geographic distance and/or be service connected, particularly for mental health conditions such as PTSD [4, 5]. This may also be a function of limited access to such care, especially in rural-serving non-VHA clinics which often are low-resource and located in mental health professional shortage areas. FQHC only veterans had higher frequencies of tobacco use disorder, obesity, and infectious diseases which may be related to different methods of screening between systems and/or targeted focus on certain conditions and possibly reflects veterans use of the nearby FQHC for primary care needs (e.g., upper respiratory tract infections were very common). As noted, this FQHC was > 40 miles from the nearest VHA point of care so such utilization is reasonable [5].

Broadly, these findings may inform future VHA-community care partnerships but more research on healthcare utilization is needed as non-enrolled veterans may seek enrollment and obtain access via the MISSION Act. Caution is needed as we were not able to determine distance to the nearest VHA facility or exemption from copayments for VHA care, which are associated with dual use. Moreover, causality for differences in illness patterns between the two systems also should not be inferred. Future partnerships between FQHC and VHA facilities would benefit from efforts to identify non-enrolled veterans presenting for community care who may gain access to specific VHA services such as mental health [2]. Efforts by community-based clinics to increase screening and care specifically for PTSD, substance use, and sleep disorder may also benefit veterans.

## Supplementary Information

**Additional file 1: Table S1.** Demographics of mutually exclusive samples of veterans accessing VHA and/or FQHC care*

## Data Availability

Not applicable.

## Abbreviations

FQHC: Federally Qualified Healthcare Centers
ICD-10: International Classification of Diseases, Tenth Revision
PTSD: Post-traumatic stress disorder
VHA: Veterans Health Administration
