# Supplementary material for: Veterans utilizing a federally qualified health center: a clinical snapshot
Source: Mil Med Res. 2022 Apr 13;9:18. doi: 10.1186/s40779-022-00379-y (PMC9006461; doi:10.1186/s40779-022-00379-y)
Supplement: Supplementary file 1 — Additional file 1: Table S1. Demographics of mutually exclusive samples of veterans accessing VHA and/or FQHC care* [file 40779_2022_379_MOESM1_ESM.pdf]

Table S1 Demographics of mutually exclusive samples of veterans accessing VHA and/or FQHC care*

Characteristic	FQHC use only veterans (n = 433)	Dual use veterans (n = 349)	P-value
Age (Mean ± SD)	56 ± 18.1	60 ± 15.6	0.0002
Gender [n(%)]			
Male	374 (86.4)	321 (92.0)	0.01
Female	59 (13.6)	27 (7.7)	0.009
Unknown	0 (0.0)	1 (0.3)	-
Race [n(%)]			
Black	22 (5.1)	15 (4.3)	0.61
White	384 (88.7)	258 (73.9)	< 0.0001
Other	3 (0.7)	8 (2.3)	0.056
Unknown	24 (5.5)	68 (19.5)	-
Ethnicity [n(%)]			
Hispanic or Latino	42 (9.7)	3 (0.9)	< 0.0001
Not Hispanic or Latino	376 (86.8)	278 (79.6)	0.007
Unknown	15 (3.5)	68 (19.5)	-

*Comparisons were conducted using t-test for averages and chi-square with significance set at $P < 0.05$. *FQHC* Federally Qualified Healthcare Centers, *VHA* Veterans Health Administration
